# AlGaN Nanowires for Ultraviolet Light-Emitting: Recent Progress, Challenges, and Prospects

**DOI:** 10.3390/mi11020125

**Published:** 2020-01-23

**Authors:** Songrui Zhao, Jiaying Lu, Xu Hai, Xue Yin

**Affiliations:** Department of Electrical and Computer Engineering, McGill University, 3480 University Street, Montreal, QC H3A 0E9, Canada; jiaying.lu@mail.mcgill.ca (J.L.); xu.hai@mail.mcgill.ca (X.H.); xue.yin@mail.mcgill.ca (X.Y.)

**Keywords:** compound semiconductor, nanostructure, ultraviolet, light-emitting diode (LED), molecular beam epitaxy, GaN, AlN

## Abstract

In this paper, we discuss the recent progress made in aluminum gallium nitride (AlGaN) nanowire ultraviolet (UV) light-emitting diodes (LEDs). The AlGaN nanowires used for such LED devices are mainly grown by molecular beam epitaxy (MBE) and metalorganic chemical vapor deposition (MOCVD); and various foreign substrates/templates have been investigated. Devices on Si so far exhibit the best performance, whereas devices on metal and graphene have also been investigated to mitigate various limitations of Si substrate, e.g., the UV light absorption. Moreover, patterned growth techniques have also been developed to grow AlGaN nanowire UV LED structures, in order to address issues with the spontaneously formed nanowires. Furthermore, to reduce the quantum confined Stark effect (QCSE), nonpolar AlGaN nanowire UV LEDs exploiting the nonpolar nanowire sidewalls have been demonstrated. With these recent developments, the prospects, together with the general challenges of AlGaN nanowire UV LEDs, are discussed in the end.

## 1. Introduction

Compared with bulk materials, low-dimensional materials such as nanowires can have different electrical and optical properties, such as the strong confinement of charge carriers and photons associated with the reduced dimensions. Motivated by exploring novel electrical and optical properties at low dimensions as well as new material platforms for future generation electronic and photonic devices, tremendous efforts have been devoted in the past two decades to the study of semiconductor nanowires and their device applications; and remarkable progresses have been made in applying various semiconductor nanowires to light-emitting devices, solar energy conversion devices, transistors, and biosensors [1,2,3,4,5,6,7,8,9,10,11,12,13,14,15,16,17]. 

Among various semiconductor nanowires, aluminum gallium nitride (AlGaN) nanowires, due to their direct and tunable bandgap energies from ~3.4 eV to 6 eV (corresponding to ~207–364 nm), are of particular interest for mid-deep ultraviolet (UV) light-emitting diodes (LEDs) and lasers [18]. Semiconductor UV light-emitting technologies are positioned to replace conventional UV light-emitting technologies, which are predominantly relying on mercury lamps, for a wide range of applications, such as water disinfection, curing, sensing, to name just a few [18,19,20,21].

Besides the suitable bandgap energies, there are a few other important reasons to investigate AlGaN nanowire structures for UV light-emitting: (1) In nanowire structures, due to the large surface to the bulk volume ratio, the lattice strain, due to the lattice mismatches between AlGaN and commonly used substrates as well as between GaN and AlN, can be better accommodated compared with planar counterparts, promising a better material quality [22]; (2) In nanowire structures, the doping concentration can be higher than that in planar counterparts due to the enhanced surface doping [23,24,25,26], which could be highly beneficial for the electrically injected light-emitting devices; and (3) The possibility of having devices on different substrates, including flexible substrates, through an *in-situ* integration [27,28,29].

The past few years have witnessed the rapid development of AlGaN nanowire UV LEDs, as well as lasing under the direct electrical injection [30,31,32,33,34,35]. For example, AlGaN nanowire deep UV LEDs with milli- to sub-milli-watt light output power have been demonstrated [36,37]. These devices are made viable due to the use of high-quality AlGaN nanowires grown by molecular beam epitaxy (MBE), the improved p-type doping with the use of nanowire structures, the use of tunnel junction to improve the carrier injection (in particular the hole injection), and the presence of high Al content AlGaN passivation shell that can confine the charge carriers in the nanowire bulk region [36,37]. In this review paper, we discuss the recent progress made in AlGaN nanowire UV LEDs. 

This paper is organized as follows: Section 2 presents a brief overview of various AlGaN nanowire synthesis techniques, with a focus on the bottom-up approach. It is noted, though, AlGaN nanowires can also be obtained through the top-down etching, e.g., Ref. [38]. As the majority device studies are on Si substrate, following Section 2 we first discuss the recent progress of devices on Si in Section 3, focusing on AlGaN nanowire tunnel junction deep UV LEDs, which show the state of the art performance for large-area devices on Si; and such devices also have much better performance compared with large-area devices on other foreign substrates. Given various limitations of Si substrate, such as the UV light absorption, in Section 4 we discuss some possible solutions using other foreign substrates/templates, including metal and graphene; and we again highlight the best performance achieved so far. We then move on to AlGaN nanowire UV LEDs with nanowires grown on patterned substrates in Section 5, aiming to show some possible solutions to issues related to the spontaneously formed nanowires. In Section 6, we discuss nonpolar AlGaN nanowire quantum well UV LEDs, exploiting the nonpolar sidewalls of the wurtzite nanowire structure. This represents an alternative approach to reduce the quantum confined Stark effect (QCSE) in AlGaN quantum well UV LEDs, in addition to the use of challenging nonpolar/semipolar substrates [39]. The prospects, together with the general challenges of AlGaN nanowire UV LEDs, are discussed in Section 7. 

## 2. A Brief Overview of Synthesis Techniques

A wide range of techniques have been explored to synthesize AlGaN ternary nanowires (including AlN nanowires). The detailed growth studies can be found in a number of review papers [15,30,32,34]. In what follows, we briefly discuss the major synthesis techniques for AlGaN ternary nanowires.

### 2.1. CVD and PVD

Chemical vapor deposition (CVD) and Physical vapor deposition (PVD) typically involve precursors and carrier gases, with or without catalysts. The growth is generally described by the vapor–liquid–solid (VLS) mechanism [40,41,42,43]. Using these techniques, AlGaN nanowires with a wide range of Al contents (from 0 to 100%) have been achieved. Nonetheless, AlGaN nanowires synthesized by these techniques typically emit light in the near UV and/or visible spectral ranges due to defects, making device development challenging. 

### 2.2. MBE and MOCVD

Hitherto, large-area AlGaN nanowire UV LEDs are mainly fabricated using AlGaN nanowires grown by epitaxy tools, including MBE and metalorganic chemical vapor deposition (MOCVD, also called metalorganic vapor phase epitaxy, MOVPE); and the shortest wavelength with AlGaN ternary nanowires is 236 nm [44], whereas 207 nm emission has been achieved using AlN nanowires [25,45]. The early efforts of growing AlGaN nanowires using such large-scale epitaxy tools can be dated back to around 2000, when AlGaN nanowires with low Al contents were first investigated by MBE [46,47]. These early efforts were followed by tremendous efforts from a large number of groups who have been working on the epitaxial growth of AlGaN nanowires (primarily by MBE) [25,28,48,49,50,51,52,53,54]. In these studies, the AlGaN nanowires are typically spontaneously formed on 2-inch or 3-inch Si substrates under the nitrogen rich conditions, with the help of GaN nanowire template. These substrate sizes are mainly limited by reactor design, and in principle there are no fundamental limitations to scale up the growth to larger substrate sizes. The growth is generally understood through a diffusion-driven, self-organized mechanism [55,56]. Due to different chemical potentials on the nanowire top surface and the sidewall, the impinged atoms diffuse at the substrate surface and then migrate to the nanowire top, promoting a spontaneous vertical growth. Furthermore, for GaN nanowires grown by MBE, lattice registration (a requirement for the growth of epi-layers) is not needed [57], which enables the formation of an AlGaN nanowire segment on a wide range of substrates [27,28,29]. 

### 2.3. Selective Area Growth

To further improve the nanowire uniformity, AlGaN nanowires on patterned substrates have also been demonstrated [58,59,60,61,62,63]. In such a growth process, a mask layer is typically required; and the nanowire nucleation site is determined by the opening, due to the different chemistries of the impinged adatoms on the surface of the substrate and the surface of the mask material. Using such a technique, highly uniform AlGaN nanowires, across a wide range of Al contents, have been reported [60]. Alternatively, such a selective area epitaxy can also be achieved using etched GaN nanopillars [64]. It is noted, though, that in general, selective area growth can be achieved with various growth techniques, including CVD/PVD, MOCVD/MOVPE, and MBE. 

## 3. AlGaN Nanowire UV LEDs on Si

Si has been playing a dominant role in modern information and communication technologies, and it is thus of great interest in integrating light sources with Si technologies and/or on Si substrates. Further, given the low cost of Si substrate, the majority of studies of group-III nitride nanowire UV LEDs are on Si substrate. These nanowire LED structures are primarily grown by MBE (through a spontaneous formation process, as afore-discussed), and predominantly with AlGaN ternary nanowires [25,36,37,51,65,66,67,68,69]. The relatively longer history of investigating the MBE growth of AlGaN nanowires on Si, compared with the growth on other foreign substrates, has also made AlGaN nanowire UV LEDs on Si of better performance compared with devices on other foreign substrates, albeit with various limitations of using Si substrate (see Section 4). In this section, we focus on the recent advances of AlGaN nanowire deep UV LEDs on Si.

### 3.1. Basic Device Structure

**Figure 1:**
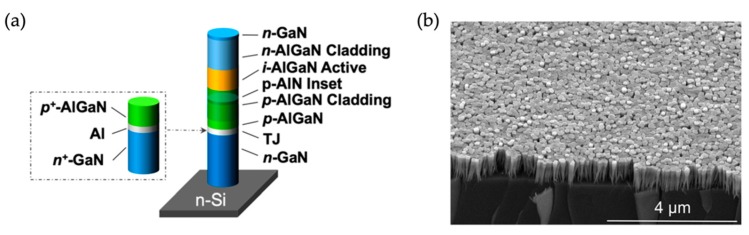
(**a**) Schematic of an individual AlGaN nanowire used for the large-area AlGaN nanowire tunnel junction deep ultraviolet light-emitting diodes (UV LEDs). (**b**) SEM image of the AlGaN nanowires at a large scale [70].

Figure 1a shows the layer-by-layer structure of an individual AlGaN nanowire that is used to form the large-area AlGaN nanowire deep UV LEDs. Figure 1b shows the SEM image of the AlGaN nanowires at a large scale. It is seen that a relatively uniform nanowire height and top-surface diameter can be achieved even if the nanowires are spontaneously formed. The device fabrication process involves photolithography and metallization [36,37]. The typical device size varies from 300 μm × 300 μm to 1 mm × 1 mm. Comparing the light emission intensity under optical pumping and electrical injection for device structures with and without the tunnel junction, a drastic improvement of light intensity under electrical injection (by more than two orders of magnitude) is measured, whereas a similar intensity is measured under optical pumping; this indicates that the improvement of light intensity under electrical injection is due to the improved carrier injection (i.e., the injection of charge carriers into the active region) [37]. 

It is noted, though, tunnel junctions involving large bandgap thin films have remained challenging to realize. The success of having the GaN-based nanowire tunnel junction is due to the enhanced dopant incorporation in nanowire structures [23,25,26,71,72].

### 3.2. Electrical Properties 

**Figure 2:**
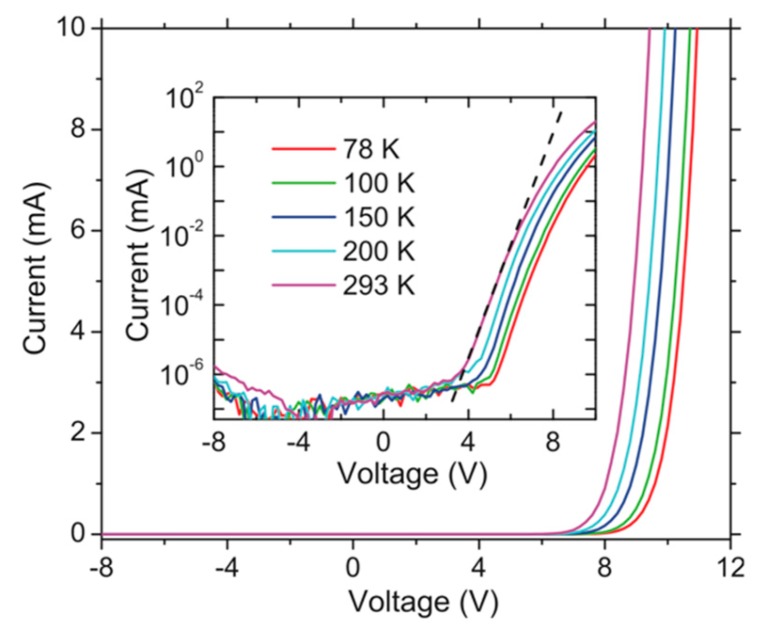
I-V characteristics of AlGaN nanowire tunnel junction deep UV LEDs. Device size: 1 mm × 1 mm [70]. The dashed line is a guide for the eye.

The detailed I-V characteristics of such AlGaN nanowire tunnel junction deep UV LEDs have also been investigated [70]. It is found that the impurity band conduction, associated with the heavily p-doped AlGaN cladding layer, plays an important role in the electrical properties. First, the deviation from the low injection regime of the diode occurs at low injection currents. As shown in the inset of Figure 2, the deviation from the low injection regime, marked by the dashed line, occurs at a relatively low injection current (~0.1 mA). This is because the impurity band conduction is typically associated with low carrier mobility [25,45,73,74], which immediately leads to a large difference between the electron mobility and the hole mobility; and this large mobility difference can lead to the deviation from the low injection regime at low injection currents [75]. Secondly, I-V characteristics are nearly temperature-independent under high injections. As shown by Figure 2, the I-V curves at different temperatures show a similar slope at a forward voltage of around 10 V. This is because, under high injections, the bottleneck for conduction is the p-AlGaN cladding layer and the conduction of the p-AlGaN cladding layer is dominated by the impurity band condition, which is associated with small active energies for electrical conduction [25,73,74]. 

### 3.3. Light-Emitting Properties

#### 3.3.1. Electroluminescence Spectra

**Figure 3:**
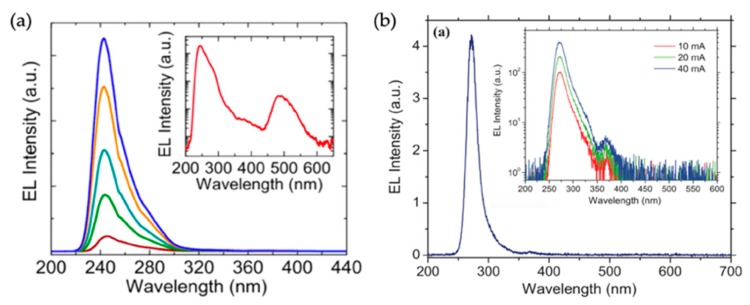
(**a**) EL spectra of AlGaN nanowire tunnel junction deep UV LEDs emitting at 242 nm under injection currents varying from 2 to 60 mA. Device size: 0.5 mm × 0.5 mm. Inset: the EL spectrum in the semi-log scale under an injection current of 20 mA. (**b**) EL spectra of AlGaN nanowire tunnel junction deep UV LEDs emitting at 274 nm under an injection current of 20 mA. Device size: 1 mm × 1 mm. Inset: EL spectra in the semi-log scale under different injection currents [37,70].

In general, for large-area AlGaN nanowire deep UV LEDs, besides the near band-edge emission peak, additional emission components have been observed [36,37,70]. This can be seen from the electroluminescence (EL) spectra of devices emitting at 242 and 274 nm in the semi-log scale (inset of Figure 3a,b; the EL spectra in the linear scale is shown in Figure 3a,b). The emission at around 320 and 380 nm for both samples could be attributed to radiative recombinations from the p-AlGaN and p-GaN layers, respectively [36,37,70], whereas the emission component at around 300 nm for both samples could be related to the localized states due to the compositional fluctuations in AlGaN nanowires grown in the nitrogen rich conditions [70].

The emission component at around 480 nm for the 242 nm emitting device is not discussed previously. Here, we suggest that it is likely related to the Al vacancy (V_Al_^3−^), as in the previously reported unintentionally and/or *n*-type doped AlGaN thin films and/or thin-film quantum wells [76,77,78]. This explanation is further supported by the absence of this emission component and/or the negligible contribution of this emission component to the entire EL spectrum (Figure 3b) for the device emitting at 274 nm, as the formation energy of V_Al_^3−^ has been suggested to increase as the Al content decreases by first principle calculations, becoming unfavorable [79,80,81,82,83,84]. Furthermore, as V_Al_^3-^ exists in the unintentionally and/or n-type doped AlGaN, it further suggests that V_Al_^3-^ presumably exists in the active region. It is also worthy of noting that, a similar defect luminescence has also been observed in AlGaN thin-film quantum wells that show more than 80% IQE [78]. 

#### 3.3.2. Light Output Power

**Figure 4:**
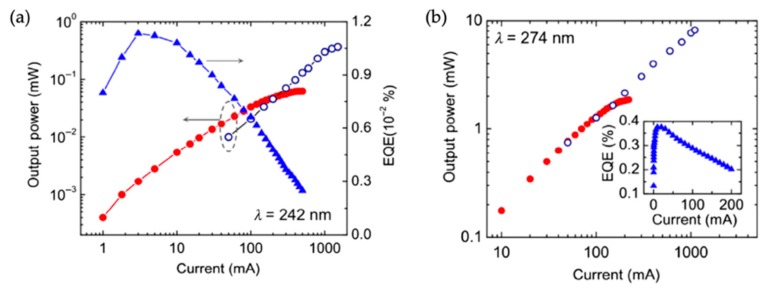
(**a**,**b**) Light output power and EQE vs. the injection current for AlGaN nanowire tunnel junction deep UV LEDs emitting at 242 and 274 nm, respectively. Device size: 1 mm × 1 mm [32]. Open symbols represent devices under the pulse operation, whereas filled symbols denote devices under the continuous-wave (CW) operation.

The light output power of devices operating at 242 and 274 nm has also been investigated in detail [36,37]. The light output power vs. the injection current under continuous-wave (CW) and pulse operations for a device emitting at 242 nm is shown in Figure 4a. It is seen that under the CW biasing, a maximum power of 0.06 mW is measured; and under the pulsed biasing a maximum power of 0.38 mW is measured, largely due to the minimization of Joule heating under the pulsed biasing. A maximum external quantum efficiency (EQE) is further derived to be ~0.012%. For devices operating at around 274 nm (Figure 4b), a maximum light output power of 8 mW is measured, with a maximum EQE of 0.4 %. These EQE numbers are within the range of typical AlGaN thin-film quantum well deep UV LEDs, i.e., ~0.04–0.2% for devices operating at around 240 nm and ~0.1–20% for devices operating at around 275 nm [19]. It is noted that the performance of the AlGaN nanowire deep UV LEDs is evaluated by measuring the light output power from the device top surface without any packaging. The use of a relatively thick top-contact metal layer (~ 20 nm) also blocks the light emission severely. Other losses could be attributed to the light absorption by the Si substrate and the light trapping effect in the spontaneously formed nanowires [85,86,87,88]. 

#### 3.3.3. Efficiency Droop

The efficiency droop has been further analyzed for devices operating at 274 nm [70]. For such AlGaN nanowire deep UV LEDs, the detailed analysis suggests that the efficiency droop occurs at a current density in the range of 0.3–3 A/cm^2^ [70]. As a large Shockley–Read-Hall (SRH) rate can overshadow the efficiency droop [89], it is thus noted that the efficiency droop onset current density for such devices could occur at an even lower current density. Further given the relatively thick active region (around 40 nm), it is thus suggested that the Auger process might not be a dominant reason for the efficiency droop. In addition, given the bifurcation current density under the CW operation and pulse operation for the light output power vs. the injection current is much higher than the efficiency droop onset current density, Joule heating is not likely playing a major role in the efficiency droop. Further detailed analysis suggests that the efficiency droop is largely due to the poor hole mobility, fundamentally associated with the impurity band conduction in highly p-doped AlGaN alloys [70]. This is also consistent with the observation that the efficiency droop occurs in the high injection regime (Figure 2). A similar efficiency droop mechanism might be applied to devices emitting at 242 nm.

## 4. AlGaN Nanowire UV LEDs on Other Foreign Substrates

Despite the progress made for devices on Si substrate, the limitations of using Si substrate are also obvious, e.g., the strong light absorption in the UV spectral range, the spontaneously formed SiN_x_ that might be a barrier for the electrical charge transport [90]. This motivates the studies of AlGaN nanowire UV LEDs on other foreign substrates, including metal and graphene. In this section, we discuss the recent development of AlGaN nanowire UV LEDs on these substrates.

### 4.1. Metal Foils and Metal-coated Substrates 

**Figure 5:**
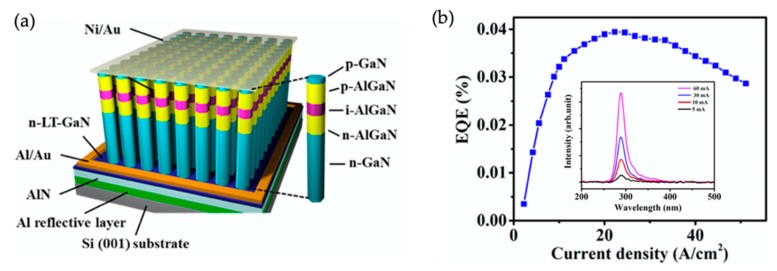
(**a**) Schematic of AlGaN nanowire deep UV LEDs on Al-coated Si substrate. (**b**) EQE vs. the injection current, with the inset showing the EL spectra under different injection currents (from 5 mA to 60 mA) [29].

Over the past few years, various metal foils (e.g., Ti, Ta) [27,91] and metal-coated substrates (e.g., Al, Pt, Ti, Mo) [28,29,92,93,94,95,96,97] have been investigated for the growth of AlGaN nanowire UV LED structures, motivated by the excellent physical properties of metals, including thermal and electrical conductivity, light reflection, as well as flexibility. In addition, by coating a metal layer to Si substrate one can also reduce the formation of SiN_x_. These LED structures are primarily grown by MBE. So far, the shortest emission wavelength of AlGaN nanowire UV LEDs with the use of a metal layer is at 288 nm [29]. Such AlGaN nanowire deep UV LEDs use a simple AlGaN p-i-n axial junction and are fabricated on Al-coated Si substrate for a better UV light reflection compared with Ti (Figure 5a). The EL spectra under different injection currents are shown in the inset of Figure 5b. A maximum EQE of ~0.04% is reported from such AlGaN nanowire deep UV LEDs (Figure 5b). 

It is seen that, despite the progress made for devices on metal foils and/or metal-coated substrates, the device performance has remained inferior to that of devices on Si. One major issue is delamination, which leads to non-uniform nanowires. The metal delamination is proposed to be associated with the different thermal expansion coefficients of the underlying substrate and the metal layer on top [94]. Another issue is associated with the crystalline form of the commonly used metal foils, i.e., polycrystalline. In general, the orientation of nanowires is strongly correlated to the microstructure of the underlying substrate; and due to the different grains associated with the polycrystalline metal foils, the nanowires tend to be tilted with respect to the c-axis (the growth direction) at a large scale, leading to the coalescence of nanowires and the metal deposition on the nanowire sidewalls that causes electrical current leakage paths and thus deteriorates the device performance [27]. Solutions using metallic glasses (amorphous) and nanocrystalline metal films have been investigated; and an improved nanowire uniformity has been achieved, due to the reduced grain size [92]. Addressing these issues could lead to a further improvement of the device performance on metal. It is also noted that, although with the above issues, a further improvement of the device performance might also be obtained by using tunnel junctions as in devices on Si (Section 3). 

### 4.2. Graphene

AlGaN nanowire UV LEDs on the graphene-coated glass substrate have been demonstrated recently [98]. The UV light is emitted through the substrate. This is made possible due to the use of UV light transparent glass substrates and the graphene bottom electrode that is not only transparent to the UV light but also possesses a low sheet resistance. 

**Figure 6:**
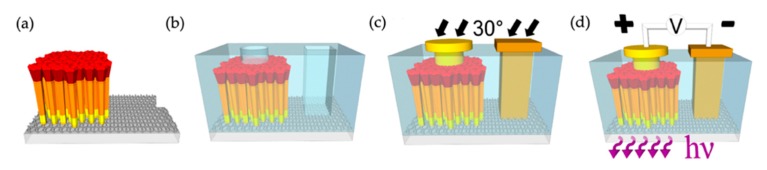
(**a**) Schematic of AlGaN nanowire UV LED structures grown on the graphene-coated glass substrate. (**b**) Openings in polymer for metal contacts to AlGaN nanowires and graphene. (**c**) Schematic of metal contacts deposition at a tilting angle of 30°. Au is used for the contact to graphene. (**d**) Schematic of light-emitting through the substrate [98].

In this work, GaN/AlGaN double heterostructures are used for light-emitting. Schematically shown in Figure 6a, such AlGaN nanowire LED structures are grown on a double-layer graphene coated amorphous silica glass by MBE. The device fabrication and the emission schematic are shown in Figure 6b–d. The electrical current injection is realized through the bottom metal contact to graphene and the top metal contact to p-type GaN. 

**Figure 7:**
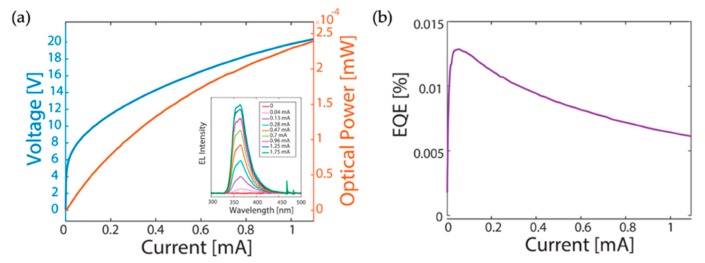
(**a**) I-V characteristics and the light output power vs. the injection current for AlGaN nanowire UV LEDs on graphene. Inset: EL spectra under different injection currents (from 0 mA to 1.75 mA). (**b**) EQE vs. the injection current [98].

The I-V characteristics of such AlGaN nanowire UV LEDs are shown in Figure 7a. It is noted that the operation voltage is quite high, which is ascribed to the drastically increased sheet resistance of the double-layer graphene after the MBE growth. Protecting the graphene layer during the MBE growth of AlGaN nanowires represents a challenge for devices on graphene. Also shown in Figure 7a is the light output power vs. the injection current for light emission around 365 nm (the emission spectra are shown in the inset of Figure 7a); and it is seen that under an injection current of 1 mA (the circular p-metal diameter is 150 μm), a light output power of ~250 nW is measured. The maximum EQE is further derived to be ~0.01% (Figure 7b). 

## 5. AlGaN Nanowire UV LEDs on Patterned Substrates

**Figure 8:**
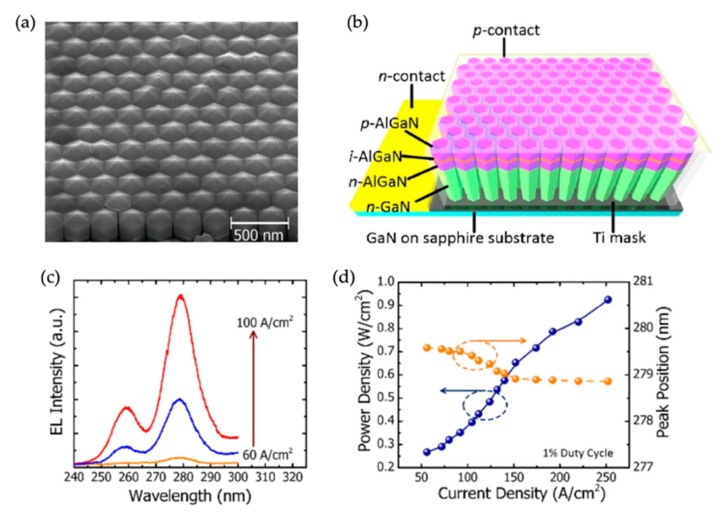
(**a**) SEM image of AlGaN nanowires grown on a patterned GaN-on-sapphire template [61]. (**b**) Schematic of AlGaN nanowire UV LEDs on such a patterned template. [Reprinted/Adapted] with permission from Ref. [60] The Optical Society. (**c**,**d**) EL spectra and the light output power vs. the injection current density, respectively. Also shown in (**d**) is the peak position vs. the injection current density. Device size: 50 μm × 50 μm. [Reprinted/Adapted] with permission from Ref. [60] The Optical Society.

For the above-discussed AlGaN nanowire UV LEDs, the nanowires are spontaneously formed. In order to further improve the device performance, it is necessary to control the nanowire size and spacing. In the past, AlGaN nanowire UV LEDs on patterned substrates using both GaN and AlGaN active regions have been reported [58,60,61]. In this section, we focus on the recent demonstration of AlGaN nanowire deep UV LEDs on patterned GaN-on-sapphire templates [60,61]. The SEM image of AlGaN nanowires on such a patterned template is shown in Figure 8a. It is seen that nanowires with highly identical size and spacing are obtained. Figure 8b shows the schematic of the fabricated device. The electrical injection is realized through the top metal contact to the p-type contact layer and the bottom metal contact to the n-type GaN template [60]. The EL emission spectra under different injection currents are shown in Figure 8c. The two peaks at around 275 and 260 nm correspond to the emission from the active region and the cladding layer, respectively [60]. For such AlGaN nanowire UV LEDs, a maximum power density of 1 W/cm^2^ is measured under an injection current density of 250 A/cm^2^ (Figure 8d). 

Using regular AlGaN nanowire arrays, a high light extraction efficiency of around 70% has also been suggested for an emission wavelength of 280 nm, through exploiting photonic bandgap effects [99]. This could greatly mitigate the light trapping effect in the spontaneously formed nanowires [85,86,87,88]. The precise control on the nanowire size and spacing could enable a rational design-to-realization of AlGaN nanowires UV LEDs; and it also opens a door to engineer the generation and propagation properties of deep UV photons. Moreover, the high nanowire uniformity could lead to highly uniform AlGaN passivation shells in each individual nanowire, improving the charge carrier confinement. This is in contrary to the case with the spontaneously formed nanowires (Section 3). In addition, through controlling the lateral growth rate, a coalesced nanowire top can also be achieved; this can largely mitigate the challenge to make the top metal contact [61]. 

## 6. Nonpolar AlGaN Nanowire UV LEDs

Due to the presence of the large electrical polarization fields in c-plane group-III nitrides, strong QCSE is present in quantum well LEDs based on c-plane group-III nitrides. Making devices on nonpolar/semipolar planes promises the reduced electrical polarization fields and thus the improved device performance [39,100,101,102,103]. Nonetheless, having high quality epitaxy-ready nonpolar/semipolar substrates/templates has remained challenging [39]. This makes using nanowire structures appealing: For group-III nitride nanowires grown either by MOCVD or MBE, the sidewalls are naturally nonpolar, due to the wurtzite structure. 

In this context, different approaches have been investigated to obtain nonpolar light-emitting regions using AlGaN nanowires. Coulon et al. have investigated the overgrowth of the nonpolar AlN/AlGaN/AlN single quantum well on the top-down etched AlN core using MOCVD, and cathodoluminescence experiments indicate a deep UV emission around 229 nm [104]. Brubaker et al. have investigated the growth of the core-shell AlGaN/GaN p-i-n UV LED structures by MBE, and demonstrated a 5× higher light-emitting intensity compared with the axial p-i-n junctions. In this work, the AlGaN shell is realized by reducing the substrate temperature [105]. 

**Figure 9:**
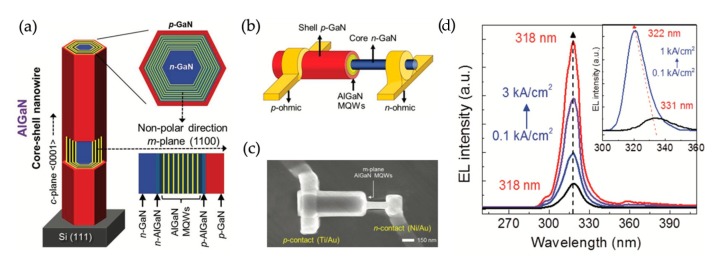
(**a**) Schematic of the structure of single AlGaN nanowire UV LEDs using lateral nonpolar quantum wells. (**b**) Schematic and (**c**) SEM image of the fabricated single AlGaN nanowire UV LED with lateral nonpolar quantum wells. (**d**) EL spectra under different injection currents. Inset: EL spectra of an axial nanowire UV LED for a comparison purpose (see the main text) [106].

Recently, single AlGaN nanowire UV LEDs using lateral quantum wells have also been reported [106]. Such single nanowire LED structures are grown by MOCVD on Si substrate. The lateral quantum wells are achieved by reducing the substrate temperature. The schematic of the device structure is shown in Figure 9a. For the device fabrication, the as-grown AlGaN nanowires are dispersed to SiO_2_-coated Si substrate, followed by a focused ion beam (FIB) etching to expose the n-GaN nanowire core. The metal contacts are realized by standard e-beam lithography and metallization processes. The electrodes are annealed at 550 ℃ for 2 min in N_2_ environment. Ti/Au (20/50 nm) and Ni/Au (20/50 nm) are used for n- and p-contacts, respectively. The schematic of the single nanowire LED and the SEM image of a fabricated device are shown in Figure 9b,c, respectively.

The EL spectra under different injection currents are shown in Figure 9d. The inset of Figure 9d shows the EL spectra measured from an axial single nanowire LED for a comparison purpose. It is seen that compared with the strong blueshift in the axial single nanowire LED, the single nanowire LED using lateral nonpolar quantum wells shows a negligible blueshift, suggesting a greatly reduced QCSE. Compared with the EL spectral linewidth of devices using ensemble nanowires (~18 nm) [36,37], the single nanowire LED using lateral nonpolar quantum wells possesses a narrower linewidth (~11 nm), allowing the examination of more detailed spectral characteristics. The EQE of such nonpolar single nanowire LEDs is further derived to be ~3% [106]. 

## 7. Conclusions and Prospects

In this paper, we have reviewed the recent progress made in AlGaN nanowire UV LEDs. Using the spontaneously formed AlGaN nanowire structures on Si, devices with optical performance comparable to conventional AlGaN quantum well UV LEDs have been achieved. Furthermore, devices on other foreign substrates have been investigated to mitigate various limitations of Si substrate. Moreover, a selective area epitaxy technique has been used to fabricate AlGaN nanowire UV LEDs, to overcome issues with the spontaneously formed nanowires. In addition, nonpolar AlGaN quantum well UV LEDs, exploiting the natural nonpolar facets (sidewalls) of wurtzite AlGaN nanowires, have been demonstrated, representing another approach to reduce the QCSE in AlGaN quantum wells. 

Further improving the performance of AlGaN nanowire UV LEDs requires solving the challenges related to nanowires, including surface defects/states, light extraction, and fabrication (in particular for the top metal contact). Nonetheless, as discussed in this paper, there are solutions readily available for these challenges. For example, Al-rich AlGaN shells can passivate the nanowire surface (and thus surface effects can be minimized); and the uniformity issue of the AlGaN shells with the spontaneously formed nanowires can be addressed by using nanowires grown by selective area epitaxy, which can give highly uniform nanowires. Moreover, with the use of transparent substrate/template, light can be extracted from the backside; and thus, in principle the light blocking by contacts is not a concern for nanowire devices. It is also noted that the light trapping effect in the spontaneously formed nanowires can also be greatly alleviated by using nanowires grown by selective area epitaxy. In addition, the coalescence of the nanowire top, in a controlled manner, has been shown by selective area epitaxy, which can greatly address the top metal contact issue. 

AlGaN quantum well UV LEDs have been investigated in the past two decades and significant progress has been made [20]. However, the development of AlGaN quantum well deep UV LEDs is hindered by two grand challenges. The first one arises from the lack of suitable substrates, which leads to large dislocation densities. Bulk AlN substrate emerges in recent years [107], nonetheless, the high price, the small substrate size, and the deep UV light absorption due to impurities of bulk AlN hold back its applications [20]. Another challenge is p-type doping. Fundamentally, to activate a p-type dopant (Mg) in high Al content AlGaN alloys, large activation energies in the range of 400–600 meV are required at room temperature, which is a major challenge for electrically injected light-emitting devices with AlGaN alloys [108,109]. If a high Mg concentration can be achieved, it is possible to reduce the activation energy for p-type conduction, e.g., through the strong doping induced band fluctuations or utilizing the impurity band. These effects have been observed in GaN [110]. For AlGaN, however, due to the elevated growth temperature compared with that of GaN (it is noted that in order to have high-quality AlGaN alloys high substrate temperatures are required), it is difficult to achieve high Mg doping concentrations, largely due to the high Mg desorption rate at high substrate temperatures. The low Mg doping concentration in high Al content AlGaN alloys limits the free holes available at room temperature. In addition, the compensation effect from n-type defect donors also limits the free hole concentration in high Al content AlGaN alloys at room temperature. For example, for the end compound AlN, the free hole concentration is only ~10^11^ cm^−^^3^ [109,111]. 

Nanowire structures, on the other hand, could be a viable approach to solve the above two grand challenges for AlGaN deep UV LEDs: the large surface-to-bulk volume ratio can relax the lattice strain efficiently to the nanowire surface, so that the bulk region can be dislocation free; the large surface-to-bulk volume ratio can also make the dopant incorporation more efficient in nanowires. For example, free hole concentrations on the order of 10^17^ cm^−3^ have been reported from AlN nanowires [73]. This leads to a drastically improved electrical performance comparing AlN nanowire LEDs to AlN thin film LEDs [25,45,109]. 

These key advantages of AlGaN nanowires, compared with AlGaN thin films, together with the recent progress made in AlGaN nanowire UV LEDs, could make AlGaN nanowires an alternative path for semiconductor UV LEDs; and the further improved performance can be expected in the near future with addressing the above issues for nanowire devices. In addition, the much-reduced dislocation density and the drastically improved p-type doping in AlGaN nanowires could also render them as a promising path for semiconductor deep UV lasers [85,86,87,88].
